# Serum Biomarkers in a Radiological Pattern of Non-Fibrotic Hypersensitivity Pneumonitis: Implications for Mechanistic Difference and Differential Diagnosis

**DOI:** 10.3390/diseases10030036

**Published:** 2022-06-27

**Authors:** Takayuki Takimoto, Yukihiro Nakamura

**Affiliations:** 1 Department of Internal Medicine, National Hospital Organization Kinki-Chuo Chest Medical Center, 1180 Nagasone-Cho, Kita-Ku, Sakai City 591-8555, Osaka, Japan; nakamura6213@gmail.com; 2 Department of Cardiology, Pulmonology, Hypertension, and Nephrology, Ehime University Graduate School of Medicine, Toon 791-0295, Ehime, Japan

**Keywords:** radiological pattern, drug-related pneumonitis, hypersensitivity pneumonitis, serum biomarker, Krebs von den Lungen 6, surfactant protein D

## Abstract

Hypersensitivity pneumonitis (HP) is a consequence of immune-mediated reactions caused by recurrent exposure to environmental agents. Recently, clinical practice guidelines for the diagnosis of HP were published and increased interest in HP. On the other hand, novel therapies have recently emerged for various diseases, and the management of drug-related pneumonitis (DRP) has become increasingly important. Among DRP, the HP pattern (DRP-HP) shows small, poorly defined centrilobular nodules with or without widespread areas of ground-glass opacity or lobular areas of decreased attenuation and vascularity. A similar radiological pattern of non-fibrotic HP can be induced, irrespective of inhalation (non-fibrotic HP) or intravenous administration (DRP-HP). However, their difference has not been well described, although the distribution of lesions in the lungs was slightly different between these two conditions. In this review, we focus on serum biomarkers of lung epithelial cells in order to investigate the difference between DRP-HP and non-fibrotic HP (common-HP). Serum levels of Krebs von den Lungen 6 (KL-6) might be relatively lower (occasionally normal) in DRP-HP than in common-HP, implying a mechanistic difference. KL-6 could be useful in discriminating between DRP and non-fibrotic HP (common type).

## 1. Introduction

HP is a consequence of immune-mediated reactions caused by recurrent exposure to overt or occult environmental agents [1]. Recently, a clinical practice guideline for the diagnosis of HP was jointly published by the American Thoracic Society (ATS), Japanese Respiratory Society, and Asociaci’on Latinoamericana del T´orax [2]. Furthermore, the CHEST Guideline and Expert Panel Report [3] increased interest in HP. Previous guidelines used a diagnostic algorithm and confidence levels for the diagnosis of HP. Based on the findings of high-resolution (HR) computed tomography (CT) scan and histopathological features, patients can have one of the following three patterns: typical HP, compatible with HP, and indeterminate for HP with diagnostic confidence based on multidisciplinary discussions. HP is classified into non-fibrotic (formerly called acute and subacute) and fibrotic (formerly called chronic) phenotypes.

Novel therapies have recently emerged for various diseases, and the management of DRP has become increasingly important [4]. CT patterns of DRP are classified into the following based on the ATS/European Respiratory Society international multidisciplinary classification of interstitial pneumonia (IP): acute interstitial pneumonia/diffuse alveolar damage pattern, HP, cryptogenic organizing pneumonia (COP), and nonspecific interstitial pneumonia (NSIP) patterns [5]. These radiological patterns are considered to correlate with typical histopathological features [6].

DRP-HP shows small, poorly defined centrilobular nodules with or without widespread areas of ground glass opacity or lobular areas of decreased attenuation and vascularity [7], which is comparable to the radiological pattern of non-fibrotic HP as previously described in the HP guideline. The reported incidence of DRP-HP varies between 2.3% and 33.3% among all cases of DRP, depending on the drug used, study, and inclusion criteria [6,8,9,10]. Recently, immunotherapy has become a standard of care in oncology [7,11], and DRP-HP induced by immune checkpoint inhibitors (ICIs) has been reported up to 16–22% of all ICI-related pneumonitis [11,12]. Thus, in terms of the clinical management of the disease, it is important to distinguish DRP-HP from other diseases, including common non-fibrotic (inhalation) or infectious diseases [10,13].

In this review, we focus on serum biomarkers of lung epithelial cells in pneumonia with the radiological pattern of non-fibrotic HP caused by intrinsic or extrinsic antigens and discuss the implications for the mechanistic difference and possibility of a differential diagnosis. 

## 2. Radiological and Histopathological Findings in DRP-HP and Non-Fibrotic HP

The difference in the radiological and histopathological findings between DRP-HP and common-HP has not been well described. The radiological findings on HRCT resemble those of DRP-HP and common-HP [2,3]. However, a slight difference has been reported. Small centrilobular nodules were fewer and septal lines were more prominent in DRP-HP than in common-HP [14,15]. 

Similarly to the radiological findings, histopathological features were not significantly different between DRP-HP and common-HP [2,16,17]. Both diseases typically include cellular IP, cellular bronchiolitis, and poorly formed non-necrotizing granulomas. Granulomas are usually located in the interstitium but they can also be observed in the airspaces. Inflammation predominantly consists of lymphocytes, with relatively few plasma cells. As a possible difference, the location of the granulomas tended to be more prevalent in the peribronchiolar interstitium in common-HP [2,18]. In contrast, perivascular and predominantly perivenular inflammation was present in 77.8% of cases of DRP due to methotrexate [17]. 

These radiological and histopathological findings suggest that the distribution of lesions in the lungs could be slightly different between these two conditions, reflecting the different routes of intravenous administration or inhalation.

## 3. Serum Biomarkers of Lung Epithelial Cells 

Krebs von den Lungen-6 (KL-6) is a high-molecular-weight sialylated MUC1 glycoprotein expressed in alveolar type II pneumocytes and bronchial epithelia. In particular, regenerating type II pneumocytes in patients with interstitial lung disease (ILD) highly express KL-6 [19]. Surfactant proteins (SP)-D and SP-A are secretory glycoproteins that belong to the collagenous family of proteins called collectin. They are similar in their molecular structure but differ in their molecular weight, molecular size, and affinity to phospholipids [20]. They are produced mainly by alveolar type II pneumocytes and have regulatory effects on innate immunity [21].

KL-6 [19], SP-D [22], and SP-A [23,24] have been proposed as biomarkers of lung epithelial cells, and they are used particularly for ILD [25]. These markers are useful for making a differential diagnosis between ILD and other types of pneumonia, assessing the disease activity and monitoring therapeutic responses in various kinds of ILD, including idiopathic pulmonary fibrosis, NSIP, collagen vascular disease-associated IP, HP, DRP, pulmonary sarcoidosis, *Pneumocystis jirovecii* pneumonia, cytomegalovirus pneumonia, and radiation pneumonitis [14,19,25,26,27,28].

As clinical serum biomarkers, the differences between KL-6, SP-D, and SP-A levels are not clearly understood. KL-6 is a cell membrane protein, whereas SP-D and SP-A are secretory proteins. KL-6 is produced by actively regenerating type II alveolar epithelial cells, whereas SP-D is produced by type II alveolar epithelial cells once they have matured and regenerated [29]. In addition, the activation of a specific enzyme is necessary to cleave KL-6 from the cell membrane, and it is thought that more severe injury must occur to increase circulating KL-6 levels [30]. These functional differences may explain the differences in serum concentrations.

## 4. Serum Biomarkers of Non-Fibrotic Hypersensitivity Pneumonitis

HP, or extrinsic allergic alveolitis, is an immune system disorder that affects the lungs. It is typically inflammation of the airspaces (alveoli) and small airways (bronchioles) within the lung caused by hypersensitivity to environmental inhaled antigens, such as fungi, bacteria, avian antigens, and feather duvets. Immune complex-mediated (type 3) and delayed (type 4) hypersensitivity reactions are involved, and chemokines, cytokines, and a cluster of differentiation (CD) 8 cytotoxic T cell responses cause tissue reactions, including macrophage activation, granuloma formation, and fibrosis [31]. For common-HP, summer-type HP refers to acute HP positive for specific immunoglobulin G antibodies against *Trichosporon asahii*. Typical HRCT patterns include a centrilobular diffuse micronodular pattern, ground-glass opacification, and mosaic attenuation, reflecting coexisting small airway disease predominantly in the upper and middle lobes. 

A high serum level of KL-6 is caused by the inhalation of an organic antigen [19,32]. However, only a few studies have shown elevated serum KL-6 and SP-D in common-HP but not fibrotic HP [33,34] (Table 1). 

## 5. Serum Biomarkers of DRP-HP

In the clinical setting, we frequently encounter DRP, with a reported rate of 2.6–5% in ILD cohorts [4] and a prevalence of 19.4 per 100,000 individuals per year [36]. The prevalence of DRP-HP caused by ICIs is up to 16–22% [11,12].

Some studies on serum biomarkers in DRP have reported that absolute levels at the onset or changes from the baseline of KL-6 can predict the clinical outcomes of DRP [14,37]. KL-6, SP-D, and SP-A can be different and not associated, depending on the situation in DRP [30,38]. There have been no reports that have focused on serum biomarkers in DRP-HP. However, data from DRP studies suggest that serum KL-6 is not significantly elevated. Ohnishi et al. reported that the serum concentrations of KL-6 in four patients with DRP-HP were all within normal limits (cutoff level: 520 U/mL), as well as eosinophilic pneumonia and organizing pneumonia patterns, at the time of their diagnosis, even in patients with a large extent of opacities [14]. In other studies, the median KL-6 was 518.5 U/mL in 10 patients [35] and 571 U/mL in seven patients with DRP-HP [8] (Table 1). Furthermore, we recently reported three cases of DRP-HP induced by nab-paclitaxel, everolimus, and nivolumab, and their serum KL-6 levels were normal [10]. These data are not comparable, because the definitions or statuses of common-HP and DRP-HP differ depending on the study. 

Taken together with the evidence on common-HP, the serum levels of KL-6 (and possibly SP-D) might be relatively lower (occasionally normal) in DRP-HP than in common-HP. Serum KL-6 and SP-D levels might reflect the different mechanisms between DRP-HP and common-HP. The primary cellular source of KL-6 and SP-D is alveolar type II pneumocytes [19,22], and their concentrations are estimated to be extremely high in the epithelial lining fluid [22,32,39]. Therefore, an increase in the serum levels of these proteins is considered to be due to increased production by regenerating type II pneumocytes, impairment of epithelial cells by lymphocytic alveolitis, and enhanced permeability after the destruction of the air–blood barrier in the affected lungs [14,25,34,40]. Accordingly, the effect on type II pneumocytes might be different between DRP-HP and common-HP because of the entry route of the antigen. Further investigation, especially on the mechanism underlying DRP-HP, is required.

## 6. Conclusions

We should recognize that a similar radiological pattern of non-fibrotic HP can be induced, irrespective of inhalation or intravenous administration. Serum KL-6 levels might be relatively lower (occasionally normal) in DRP-HP than in common-HP, implying mechanistic differences. KL-6 could be useful in discriminating between DRP and non-fibrotic HP (common Type).

## Figures and Tables

**Table 1:** Literature review on drug-related pneumonitis with radiologic non-fibrotic HP pattern compared to non-fibrotic hypersensitivity pneumonitis.

	KL-6 (U/mL)		SP-D (ng/mL)		SP-A (ng/mL)		
	**Median**	**Range**	**Median**	**Range**	**Median**	**Range**	
non-fibrotic HP							
n = 7	2996	648–5373	N.A.	N.A.	N.A.	N.A.	Nakajima, 1998 (Ref. [33])
n = 12	2635	N.A.	N.A.	N.A.	N.A.	N.A.	Ohnishi, 2014 (Ref. [28])
n = 35	2710	1510–5700	338	180–725	N.A.	N.A.	Okamoto, 2015 (Ref. [34])
DRP-HP							
n = 4	Normal (<520)	Normal (<520)	N.A.	N.A.	N.A.	N.A.	Ohnishi, 2003 (ref. [14])
n = 7	571	215–2530	N.A.	N.A.	N.A.	N.A.	Takatani, 2008 (Ref. [8])
n = 10	518.5	264–3697	186.5	56.2–532	98.5	47.1–310	Kakugawa, 2013 (Ref. [35])
n = 3	342	222–398	177.7	72.6–261.9	98.8	72.2–112.1	Nakamura, 2021 (Ref. [10])

Krebs von den Lungen-6; SP-D, surfactant protein-D; SP-A, surfactant protein-A; HP, hypersensitivity pneumonitis; DRP-HP, drug-related pneumonitis with radiologic non-fibrotic HP pattern; N.A., not available.

## Data Availability

Not applicable.

## Abbreviations

ATS	American Thoracic Society
COP	Cryptogenic organizing pneumonia
CD	Cluster of differentiation
CT	Computed tomography
DRP	Drug-related pneumonitis
HP	Hypersensitivity pneumonitis
HR	High-resolution
ICI	Immune checkpoint inhibitors
ILD	Interstitial lung disease
IP	Interstitial pneumonia
KL	Krebs von den Lungen
NSIP	Nonspecific interstitial pneumonia
SP	Surfactant protein
